# Assessing multidimensional fidelity in a pilot optimization trial: A process evaluation of four intervention components supporting medication adherence in women with breast cancer

**DOI:** 10.1093/tbm/ibae066

**Published:** 2024-12-05

**Authors:** Sophie M C Green, Christopher D Graham, Michelle Collinson, Pei Loo Ow, Louise H Hall, David P French, Nikki Rousseau, Hollie Wilkes, Christopher Taylor, Erin Raine, Rachel Ellison, Daniel Howdon, Robbie Foy, Rebecca E A Walwyn, Jane Clark, Catherine Parbutt, Jo Waller, Jacqueline Buxton, Sally J L Moore, Galina Velikova, Amanda J Farrin, Samuel G Smith

**Affiliations:** Leeds Institute of Health Sciences, University of Leeds, Leeds LS2 9NL, UK; Department of Psychological Sciences & Health, University of Strathclyde, Glasgow G1 1QE, UK; Leeds Institute of Clinical Trials Research, University of Leeds, Leeds LS2 9NL, UK; Leeds Institute of Clinical Trials Research, University of Leeds, Leeds LS2 9NL, UK; Leeds Institute of Health Sciences, University of Leeds, Leeds LS2 9NL, UK; Manchester Centre for Health Psychology, University of Manchester, Manchester M13 9PL, UK; Leeds Institute of Clinical Trials Research, University of Leeds, Leeds LS2 9NL, UK; Leeds Institute of Clinical Trials Research, University of Leeds, Leeds LS2 9NL, UK; Leeds Institute of Clinical Trials Research, University of Leeds, Leeds LS2 9NL, UK; Leeds Institute of Health Sciences, University of Leeds, Leeds LS2 9NL, UK; Leeds Institute of Clinical Trials Research, University of Leeds, Leeds LS2 9NL, UK; Leeds Institute of Health Sciences, University of Leeds, Leeds LS2 9NL, UK; Leeds Institute of Health Sciences, University of Leeds, Leeds LS2 9NL, UK; Leeds Institute of Clinical Trials Research, University of Leeds, Leeds LS2 9NL, UK; Department of Clinical and Health Psychology, Leeds Teaching Hospitals NHS Trust, Leeds LS9 7TF, UK; Medicines Management and Pharmacy Services, Leeds Teaching Hospitals NHS Trust, Leeds LS9 7TF, UK; Wolfson Institute of Population Health, Queen Mary University of London, London, UK; Independent, Leeds LS2 9NL, UK; Independent, Leeds LS2 9NL, UK; Leeds Institute of Medical Research at St James’s, University of Leeds, St James’s University Hospital, Leeds LS9 7TF, UK; Leeds Cancer Centre, Leeds Teaching Hospitals NHS Trust, St James’s University Hospital, Leeds LS9 7TF, UK; Leeds Institute of Clinical Trials Research, University of Leeds, Leeds LS2 9NL, UK; Leeds Institute of Health Sciences, University of Leeds, Leeds LS2 9NL, UK

**Keywords:** process evaluation, intervention fidelity, optimization trial, breast cancer, medication adherence, multiphase optimization strategy

## Abstract

Adherence to adjuvant endocrine therapy in women with breast cancer is low. We conducted a 2^4-1^ fractional factorial pilot optimization trial to test four intervention components supporting medication adherence [text messages, information leaflet, acceptance and commitment therapy (ACT), self-management website], in the preparation phase of the multiphase optimization strategy. Guided by the National Institute of Health Behavior Change Consortium fidelity framework, we investigated fidelity of design, training, delivery, receipt, and enactment of four intervention components. Women prescribed adjuvant endocrine therapy (*n* = 52) were randomized to one of eight experimental conditions comprised of combinations of the four intervention components (ISRCTN: 10487576). We assessed fidelity using self-report data (4 months post-randomization), trial data, ACT session observations, behavior change technique (BCT) coding, and interviews with participants (*n* = 20) and therapists (*n* = 6). Design: Each intervention component targeted unique behavior change techniques with some overlap. Training: All 10 therapists passed the competency assessment. Delivery: All leaflets (27/27) and website (26/26) details were sent, and ACT procedural fidelity was high (85.1%–94.3%). A median of 32.5/41 (range 11–41) text messages were delivered, but a system error prevented some messages being sent to 22 of 28 participants. Receipt: Most participants [63.0% (ACT, leaflet) to 71.4% (text messages)] read all or at least some of the intervention components they were randomized to receive. Enactment was reported most positively for ACT. All intervention components demonstrated adequate fidelity. We have provided an exemplar for assessing fidelity using the National Institute of Health Behavior Change Consortium framework in the preparation phase of multiphase optimization strategy.

## Teaser

A multimethods process evaluation to assess the multidimensional fidelity of four intervention components aimed at supporting medication adherence in women with breast cancer.

**Implications**

**Practice:** Text messages, an information leaflet, acceptance and commitment therapy–based guided self-help, and a side-effect management website can be delivered with fidelity; a combination of these intervention components could support women with breast cancer to adhere to adjuvant endocrine therapy, subject to further evaluation.

**Policy:** Multidimensional assessments of fidelity, alongside effectiveness, can assure policymakers that future trial evidence will be based on intervention components that can be delivered with adequate fidelity.

**Research:** The National Institute of Health Behavior Change Consortium fidelity framework is a useful approach for comprehensively assessing fidelity in the preparation phase of the multiphase optimization strategy, to inform adaptations prior to an optimization randomized controlled trial.

## Introduction

Adjuvant endocrine therapy (AET) is prescribed to women with Stage I–III estrogen receptor–positive breast cancer (around 70%–80% of all breast cancers) for 5–10 years, to reduce breast cancer recurrence and mortality [1, 2]. AET is an oral tablet taken once daily. However, up to three quarters of women do not take AET as prescribed [3, 4], which increases risk of recurrence and mortality [5–7]. Barriers to AET adherence include forgetfulness, low beliefs about the necessity of AET and high concerns, psychological distress, and experiencing unpleasant side effects [8–10]. A multicomponent intervention is needed to target the range of barriers to adherence [8–10]. Existing interventions have shown mixed effectiveness in single or parallel group randomized controlled trials [11–14]. These designs do not provide information about the individual and combined effects of intervention components. Understanding the effects of individual intervention components and how they interact with one another could advance our understanding of how to support AET adherence [14].

The multiphase optimization strategy (MOST) proposes an optimization phase of research between preparing and evaluating an intervention [15]. In an optimization randomized controlled trial (ORCT), efficient, fully powered experimental designs are used to estimate the individual and combined effects of candidate intervention components [15, 16]. The optimal combination of intervention components can be selected, considering relevant resource constraints such as cost or time [17, 18]. The process of optimization can accelerate our understanding of multicomponent interventions, balancing intervention effectiveness with efficiency, affordability, scalability, and equity.

In the preparation phase of our “Refining and Optimizing a behavioral intervention to Support Endocrine Therapy Adherence” (ROSETA) program, we developed a conceptual model of a multicomponent intervention to target key barriers to support AET adherence: short message service (SMS) messages to reduce forgetfulness of taking AET, an information leaflet to increase necessity beliefs and reduce concerns, a self-management website to support management of side effects, and a guided self-help therapy program based on acceptance and commitment therapy (ACT) to increase psychological flexibility and reduce psychological distress (conceptual model available in Supplement 1). To assess the feasibility of undertaking an ORCT, we conducted a pilot optimization trial, which aimed to establish recruitment and retention rates, component adherence, feasibility of collecting outcome and process data; explore signals of efficacy; estimate costs of delivering each intervention component; and explore experience of participating in the trial [19].

We embedded a process evaluation in this pilot optimization trial to maximize learning [20]. The UK Medical Research Council recommends assessing fidelity in a pilot trial within a process evaluation, to assess how well an intervention is put into practice as intended [21]. Assessments of fidelity often solely focus on whether an intervention has been delivered and fail to consider other aspects such as whether participants receive, comprehend, and engage with the intervention [22, 23]. A comprehensive assessment of multiple dimensions of fidelity provides the opportunity to improve fidelity prior to an ORCT [23–25]. Improving fidelity can increase confidence that any effect observed in an ORCT is due to the intervention components themselves [23].

In this process evaluation of a pilot ORCT, we aimed to establish the fidelity of the intervention components across the five dimensions of fidelity recommended by the National Institute of Health Behavior Change Consortium (NIH BCC) [26]. Our objectives were (i) to establish which behavior change techniques (BCTs) the intervention components target and to what extent the components were distinct in terms of BCTs targeted (design), (ii) to establish the adequacy of therapist training to deliver the ACT component (training), (iii) to establish whether each intervention component was delivered as planned (delivery), (iv) to evaluate if participants received the components they were allocated to (receipt), and (v) to understand how participants used the interventions (enactment).

## Methods

### ROSETA pilot optimization trial design

The ROSETA pilot trial used a 2^4-1^ fractional factorial design to assess the feasibility of undertaking a fully powered ORCT [19]. Four candidate intervention components (SMS messages, information leaflet, guided self-help ACT, and self-management website) were operationalized as factors with two levels: “on” or “off.” Participants were randomized to one of eight experimental conditions. Each condition comprised unique combinations of the factor levels, in addition to usual care (Table 1) [19].

**Table 1:** Experimental conditions in ROSETA pilot optimization trial

Condition	Usual care	SMS	Information leaflet	ACT	Website	Randomized, *n* = 52	Interviewed, *n* = 20
1	Yes	Yes	Yes	Yes	Yes	8	1
2	Yes	Yes	Yes	No	No	7	4
3	Yes	Yes	No	Yes	No	7	3
4	Yes	Yes	No	No	Yes	6	2
5	Yes	No	Yes	Yes	No	6	3
6	Yes	No	Yes	No	Yes	6	1
7	Yes	No	No	Yes	Yes	6	3
8	Yes	No	No	No	No	6	3

Table taken directly from Green SMC, Rousseau N, Hall LH *et al.* Acceptability of four intervention components supporting medication adherence in women with breast cancer: a process evaluation of a fractional factorial pilot optimization trial. *Prev Sci* 2024;**25**:1065–78. https://doi.org/10.1007/s11121-024-01711-9.

### Candidate intervention components

The four intervention components, described below, were developed using intervention mapping [8].

#### SMS messages (target: habits)

Forty-one brief SMS messages were sent across a period of 4 months to encourage medication-taking habits. The messages were based on six BCTs theorized to support habit formation: adding objects to the environment, restructuring the physical environment, prompts/cues, action planning, habit formation, and self-monitoring of behavior [27]. Messages were sent daily for 2 weeks (14 messages), twice weekly for 8 weeks (16 messages), and then weekly for 6 weeks (6 messages), in addition to an opening message, one closing message, and three reminders that they could opt out at any time.

#### Information leaflet (target: medication beliefs)

A six-page information leaflet was sent to participants immediately after randomization. The aim of the leaflet was to increase beliefs about the necessity of AET (e.g. to prevent recurrence and mortality) and to reduce common concerns about AET (e.g. worry about ability to cope with side effects). The leaflet included information about how to take AET, visual graphics about the benefits of AET, a detailed table summarizing the prevalence of side effects of AET, diagrams explaining AET mechanisms, answers to common concerns (e.g. I don’t want to get another type of cancer), and quotes from breast cancer survivors about their motivations for taking AET [28].

#### Website (target: side effects)

Access was provided to a website containing strategies to self-manage the most common side effects of AET (hot flushes, joint pain, sleep difficulties, fatigue, vulvovaginal symptoms, nausea, and gastrointestinal problems). For each management strategy, the strength of evidence was described, informed by an umbrella review of systematic reviews and clinical guidelines on self-management strategies for common AET side effects [29]. The website also included videos from women taking AET speaking about their experiences taking AET and links to further support.

#### ACT (target: psychological flexibility and distress)

A guided self-help program aimed to increase psychological flexibility and reduce psychological distress. Four modules were based on four ACT skills: (i) mindfulness to improve awareness of the here-and-now, (ii) unhooking from unhelpful thoughts, (iii) following values to consistently choose to move in meaningful directions, and (iv) living beyond labels to take a perspective beyond labels and respond to oneself in ways that encourage growth. Each module was comprised of a participant booklet, audio files, and home-practice tasks. Modules were supported by five remote reflection sessions with a therapist: a 15-min introductory session in which the therapist introduced Module 1; three 25-min sessions following Modules 1–3, in which the content of each module was reflected on; and a closing 15-min session following Module 4. After each of the first four sessions, the therapist emailed the participant the materials for the next module, which the participant worked through at their own pace until the next session.

### Participants

#### Trial participants

Adult women with Stage I–IIIa breast cancer who had been prescribed AET for breast cancer (tamoxifen, raloxifene, anastrozole, letrozole, or exemestane) and had finished hospital-based treatment in the last 12 months were randomized in the pilot optimization trial (full inclusion criteria are available in Supplement 2). Participants were recruited from five hospitals across the UK. Participants were identified by three routes: (i) screening prior to clinic visits and approached during visit, (ii) patients who self-referred to their care team to discuss AET side effects or difficulties with adherence approached during visit, and (iii) retrospective screening of healthcare records for patients who had completed treatment in the past 12 months approached via postal invitation. A research nurse confirmed eligibility and recorded informed consent for all interested patients, either in person or via phone.

#### Trial therapists

ACT therapists were Health and Care Professions Council (HCPC) or United Kingdom Council for Psychotherapy (UKCP)-registered practitioner psychologists. All therapists received two-half days of training from a clinical psychologist with expertise in ACT. Therapists were allocated non-randomly to patients based on therapist availability.

### Process evaluation design

Guided by the NIH BCC fidelity framework, quantitative and qualitative methods were used to establish fidelity of design, training, delivery, receipt, and enactment [20, 26].

#### Fidelity of design

One author (S.G.) coded the presence or absence of BCTs in each intervention component using the behavior change technique taxonomy version 1 (BCTTv1) [30, 31]. Codes were discussed and finalized with the research team (S.S., L.H., and C.G.) [8]. Two external independent coders also coded the four intervention components. The external coders had completed the BCTTv1 online training, had between 4 and 5 years experience related to behavior change, and had previous experience coding interventions using the BCTTv1. Disagreements in coding were discussed in a half-day remote meeting with one author (S.G.) and the independent coders. For each disagreement, the BCT was either added or removed from the code list; intervention content was not changed. Where agreement could not be reached, the decision was based on the majority. The two independent coders were compensated.

#### Fidelity of training (competency of delivery)

The ACT component was the only intervention component which required training. To assess therapist competency, the ACT trainer assessed each therapist’s first session recording and evaluated it using the ACT Fidelity Measure (ACT-FM) [32]. The number of booster sessions required by therapists to support their delivery of ACT was recorded. Semi-structured interviews with therapists explored experiences and adequacy of the training to deliver the ACT component.

#### Fidelity of delivery

Automated data for the SMS component identified whether the message had been sent by the online system. Participant opt-outs and withdrawals were also recorded. For the information leaflet and website components, staff at the trial sites recorded whether the leaflet and website login details had been sent. For the ACT component, therapists completed a procedural fidelity checklist following each session, identifying which key tasks were delivered within the session were delivered. An external reviewer, who was a practitioner with ACT expertise, reviewed 10% of all 25-min ACT sessions (Sessions 2, 3, or 4) to assess for model fidelity using the ACT-FM. Semi-structured interviews with ACT therapists further explored fidelity of delivery.

#### Fidelity of receipt

Participants were asked, via questionnaires, which intervention components they recalled receiving and self-reported their engagement with the intervention components they were randomized to receive, with reasons for nonengagement. Website tracking data were collected to record the number of logins, time spent on the website, and videos watched. ACT component therapists reported on session attendance and perceived participant engagement with each module. Semi-structured interviews with participants explored receipt of the components, and interviews with ACT therapists explored perceptions of receipt of the ACT component.

#### Fidelity of enactment

Semi-structured interviews with participants explored the extent to which participants used the intervention components and any barriers to enactment. For each component participants were randomized to receive, they were asked about any use of the component and any barriers to use.

### Process evaluation procedure

The following describes the process evaluation procedure.

#### Quantitative assessments

All participants were asked to complete questionnaires at 4 months post-randomization. Nonrespondents were prompted after 1 and 2 weeks.

#### Qualitative assessments

Consenting participants were contacted to schedule an optional interview for 4 months post-randomization. Participants were asked to provide written or telephone consent. Nonrespondents were contacted after 1 week.

All consenting trial ACT therapists were asked to complete an end-of-trial interview 1 month before the end of the intervention delivery period. Nonrespondents were contacted after 2 weeks. Interview schedules for participants and therapists are available at DOI 10.17605/OSF.IO/FP8ZW.

Interviews were conducted via Microsoft Teams or telephone by one author (S.G.). All interviews were recorded via Dictaphone or MS Teams inbuilt recording. We initially planned to use assessment of information power to determine when to cease data collection [33], but as the pilot trial recruited a lower number of participants than planned, all consenting participants were invited to interview.

### Process evaluation measures

#### Receipt of intervention components

A one-item assessment asking participants to report which intervention components they received during the trial. All components were present within the response options available to participants.

#### Adherence to intervention components

For each component a participant was randomized to receive, they were asked how much of the component they read: “all of it,” “some of it,” or “none of it.” Participants randomized to the ACT component were additionally asked two questions about how much of the home-practice tasks and audio files they listened to: “none,” “at least some,” and “all of them.”

#### ACT-FM therapist stance subscale [32]

A 7-item subscale of the ACT-FM was used to assess therapist fidelity to ACT principles. Four items focus on ACT-consistent behaviors and three on ACT-inconsistent behaviors, each scored on a 4-point scale. A score of >4 on ACT-consistent behaviors (possible range 0–12) and <5 on ACT-inconsistent behaviors (possible range 0–9) was determined *a priori* to be considered competent.

#### Procedural fidelity checklist

A checklist was used to assess the completion of core intervention procedures in each ACT session. Sessions 1 and 2 included eight items, Session 3 had seven items, Session 4 had six items, and Session 5 had four items. For each session, the score achieved was divided by the maximum possible score and then multiplied by 100 to create a percentage score.

#### Therapist reported engagement with ACT module materials

For each ACT session, therapists were asked to report the extent they felt the participant had engaged with the module materials, including the manual, home-practice tasks, and audio files.

### Process evaluation analyses

#### Quantitative analysis

##### Fidelity of design

A first-order agreement coefficient statistic (AC1) was calculated to estimate the inter-rater reliability between the coders, overall for each component, and for each group of BCTs (as grouped in the BBCTTv1) [34]. Pre-established AC1 thresholds were used to define reliability: <0.2 = poor; 0.2 ≤ 0.4 = fairly poor; >0.4 ≤ 0.6 = moderate; >0.6 ≤ 0.8 = good; and >0.8 and ≤1 = very good [35].

##### Fidelity of training, delivery, and receipt

Descriptive statistics summarized the quantitative assessments.

#### Qualitative analysis

Rapid qualitative analyses were undertaken for the semi-structured interviews to allow for adaptations prior to a planned ORCT [19]. After each interview, the interviewer completed a rapid assessment procedure (RAP) sheet, which summarized key findings from the interview and illustrative quotes (available at DOI 10.17605/OSF.IO/FP8ZW). The quotes were taken from autogenerated transcripts for Microsoft Teams interviews or from selective transcription of telephone interviews. All quotes were checked for accuracy by one author (S.G.). A RAP sheet was completed for each participant. Individual RAP sheets were collated into an overall RAP sheet for each intervention component to collate key findings. A separate RAP sheet was used for trial therapist data. Throughout the data collection period, four authors (S.G., S.S., L.H., and C.G.) met regularly to discuss key findings, continuation of data collection, and areas to explore in the upcoming interviews.

## Results

### Participant characteristics

Of 141 eligible patients identified, 52 (36.9%) were randomized. Reasons for not participating included declining and being unable to contact. Across eight experimental conditions, 28 patients were randomized to receive the SMS component, 27 to the information leaflet, 27 to ACT, and 26 to the website (Table 1). The mean age of participants was 55.2 years (SD = 10.8). Most (45/52, 86.5%) participants were White, and a third (17/52, 32.7%) had degree-level education or above. 21 of 52 participants (40.4%) had Stage I breast cancer, 23 of 52 (44.2%) had Stage II, and 6 of 52 (11.5%) had Stage IIIA. Most (40/52, 76.9%) participants were prescribed an aromatase inhibitor, with the remainder (12/52, 23.1%) prescribed tamoxifen. Twenty participants completed the optional interview. Of these, 10 participants had received the SMS component, 9 the information leaflet, 10 the ACT component, and 7 the website. Three interviews were with participants randomized to receive usual care only.

### Therapist characteristics

Twelve therapists were trained to deliver the ACT sessions across the five recruiting sites. Seven therapists (58.3%) provided demographic information. Of these, four were female and three were male. There were five clinical psychologists, one health psychologist, and one psychotherapist. Time working as a therapist ranged from 10 months to 9 years. All therapists reported having prior knowledge and experience of ACT. Six therapists consented to be interviewed.

### Fidelity of design

**Figure 1:**
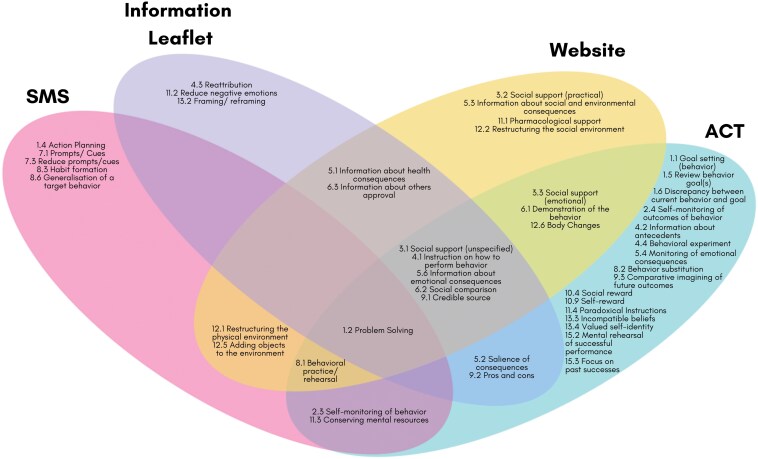
BCTs coded for each candidate component. Adapted from Green et al. [8].

Agreement of BCTs between coders ranged from good (agreement coefficient statistic 0.63, ACT) to very good (0.93, SMS). There were 67 disagreements out of a possible 372 (93 BCTs × 4 intervention components). Following discussion, 13 BCTs were added to the final code list and two BCTs were removed. Each intervention component had unique BCTs, with some overlap across components (Fig. 1).

### Fidelity of training (competency of delivery)

Twelve therapists received ACT training. Ten therapists delivered at least one ACT session and passed the competency assessment. Using the ACT-FM, the mean ACT-consistent score was 9.0 out of 12 (SD = 2.1, range = 6–12) and the mean ACT-inconsistent score was 0.9 out of 9 (SD = 1.3, range = 0–4). No therapists required a booster training session. In the interviews, therapists were positive about the training and felt adequately prepared to deliver the ACT intervention component (Table 2).

**Table 2:** Key findings from interviews with ACT therapists regarding ACT training and delivery

Fidelity dimension		Key findings
Fidelity of training	Likes	Therapists reported the training was helpful in preparing for delivery of the ACT sessions. Aspects reported as helpful were: - Experiential aspects, e.g. role plays. - Focus on the relational/ conversational aspects to improve psychological flexibility. - Mixture of teaching, interactive exercises, and opportunity for questions.
	Dislikes	One therapist felt the training was slightly too theoretical, which they felt was less necessary when delivering guided self-help.
	Suggestions for improvement	- More training on the trial elements of intervention delivery, e.g. paperwork. - More people in the training to learn from others. - Face-to-face training to improve concentration.
Fidelity of delivery	Self-efficacy of delivery	- Therapists felt confident in delivering the ACT component, which was helped with a therapist training manual to refer to, and opportunity for supervision.
	Manualized therapy	- Two therapists felt the sessions initially felt clunky to deliver due to the manualized element differing from normal clinical practice, but over time they got used to it with familiarity. - One therapist reported difficulty knowing how much deviation from the manual was appropriate.
	Length of sessions	- First session felt too short (15 min)—not enough time to understand the participant’s situation, distress and to explain ACT. - More preparation time needed for the sessions, as it was less familiar. - One therapist felt the sessions were a good amount of time to contain the participant.
	Delivery in context of NHS	- Difficulty working out who would have the time to deliver the intervention within stretched NHS resources. - Some therapists felt assistant psychologists (APs)/psychological well-being practitioners (PWPs) would be more suited to deliver guided self-help. Other therapists had concerns that APs and PWPs would not have the clinical autonomy and experience to deliver the fast-paced intervention, for example, they may struggle with the integrating psychological flexibility into conversation.
	Therapeutic relationship	- Most therapists felt there was enough time within the sessions to build a therapeutic relationship with the participant. - Therapists acknowledged the therapeutic relationship would have been more difficult to build within the time of the sessions in the participants had been more distressed. - More difficulty building rapport via phone call. - One therapist acknowledged difficulty in building a therapeutic relationship due to the teacher/pupil dynamic that can be created in guided self-help interventions. - More time in the sessions (e.g. all 25-min sessions) and more information about the participant prior to the sessions was suggested to improve the therapeutic relationship.

### Fidelity of delivery

#### SMS

Of the 41 messages intended for each participant, a median of 32.5 (range 11–41) messages were successfully delivered to the 28 participants randomized to the SMS component. Seven participants (25.0%) withdrew or opted out of receiving SMS messages, receiving a median of 18 SMS messages (range 11–37). A system error occurred during two periods in the trial, which resulted in at least one message not being sent to 22 of 28 participants (78.6%). The median number of messages not sent across all participants was six (range 0–13).

#### Information leaflet

All 27 participants randomized to receive the information leaflet component were sent an information leaflet.

#### Website

All 26 participants randomized to receive the website were sent website login details.

#### ACT

The average procedural fidelity checklist scores were 94.3% (SD = 9.7, range = 63–100) for Session 1, 85.1% (SD = 22.2, range = 38–100) for Session 2, 90.8% (SD = 14.2, range = 57–100) for Session 3, 89.2% (SD = 19.5, range = 33–100) for Session 4, and 93.8% (SD = 14.4, range = 50–100) for Session 5. The average length of sessions was 22.4 min (SD = 6.5 min). 65 of 94 session (69.1%) were conducted via telephone and 29 of 94 (30.9%) were conducted by video conferencing. Seven out of eight (87.5%) externally reviewed sessions were delivered with fidelity. The average ACT-consistent score was 9.8/12 (SD = 1.0), and the average ACT-inconsistent score was 1.0/9 (SD = 1.7). Therapists generally felt confident in delivering the ACT component, but highlighted the need for a longer first session, and questioned who would be best placed to deliver this in clinical practice (Table 2).

### Fidelity of receipt

Table 3 summarizes which intervention components participants reported receiving (with all options available to select). There were seven instances of potential contamination in which a participant reported receiving a component they were not randomized to receive, most commonly the information leaflet (four instances). There were 29 instances whereby participants did not recall receiving a component they were randomized to receive, most commonly the information leaflet (13 instances).

**Table 3:** Participant self-reported receipt of intervention components

	Reported receiving component and were randomized to receive (correct)	Reported receiving component, but not randomized to receive (incorrect)	Reported not receiving component, and not randomized to receive (correct)	Reported not receiving component, but randomized to receive (incorrect)	Missing
SMS	20	1	20	0	11
Leaflet	7	4	17	13	11
ACT	10	1	21	9	11
Website	12	1	21	7	11

Note: Receipt here refers to which components participants reported receiving, in the one-item question whereby all options were available to select from.

#### SMS

Of the 28 participants randomized to the SMS component, 16 (57.1%) reported reading all the SMS messages, 4 (14.3%) reported reading “at least some” of the messages, and 8 (28.6%) did not respond. Most interviewed participants reported they read the SMS messages and found them easy to understand. A minority of participants reported not reading all the messages, because they felt they knew what the messages were going to say. Further description and illustrative quotes from participants are available in Supplement 3.

#### Information leaflet

Of the 27 participants randomized to receive the information leaflet, 11 (40.7%) reported reading “all of it,” 6 (22.2%) read “at least some of it,” and 3 (11.1%) read “none of it.” Of those who reported reading none of the leaflet, one participant wanted to look for their own information, one had made up their mind about taking AET, and one did not want to read information about AET. Several participants in the qualitative interviews could not recall receiving the information leaflet, often citing they had received a lot of information about AET at the start of the trial. Of those who did recall receiving the leaflet, participants had generally read the leaflet at least once and found it easy to comprehend, with little “medical jargon” (see Supplement 3 for illustrative quotes).

#### Website

Of the 26 participants randomized to receive the website component, 19 (73.1%) logged in at least once. The median number of visits was 1.0 (range: 1–4). The mean time spent on the website per visit was 10.3 min (SD = 10.3). Six out of the eight videos were watched at least once. Of the pages dedicated to specific side effects, strategies to manage joint pain was viewed by most participants (11 participants) while nausea and gastrointestinal problems was viewed by the least (5 participants).

In the self-reported questionnaire, 12 of 26 (46.1%) reported having read all the information on the website, 5 of 26 (19.2%) reported reading “at least some of it,” and 2 of 26 (7.7%) reported reading none of it. Reasons for not reading any of the website information included planning to read it but not getting round to it (*n* = 1) and already receiving full information on side effects from other sources (*n* = 1).

In the qualitative interviews, most participants reported logging in at least once to the website but not revisiting the website again. Reasons for not revisiting the website included focusing on other components that they received and having no reason to revisit due to no new information being added. A minority of participants in the interviews could not recall receiving login details for the website (see Supplement 3 for illustrative quotes).

#### ACT

Of the 27 participants randomized to the ACT component, 24 (88.9%) attended Session 1, 21 (77.8%) attended Session 2, 17 (63.0%) attended Session 3, 17 (63.0%) attended Session 4, and 16 (59.3%) attended Session 5. 22 of 24 initial sessions (91.7%) took place within 4 weeks of randomization, as dictated by the protocol. Overall, two sessions were canceled with over 24 hours’ notice, eight sessions were canceled with less than 24 hours’ notice, and one session was missed without prior notice. The mean length of time between each session was between 11 (Sessions 1 and 2, SD = 5.33) and 24 days (Sessions 2 and 3, SD = 8.58).

Most participants reported reading all of the ACT module booklets (14/27, 51.9%), listening to all of the audio files (16/27, 59.3%), and completing all of the home-practice tasks (12/27, 44.4%) (Table 4). Therapists reported between 59.3% (Module 4) and 77.8% (Module 1) of participants engaged with at least some or all of the module materials (with the remainder being missing data). In the interviews, participants were positive about the flexibility of the ACT sessions and felt the module booklets were easy to understand. Barriers to receipt included sessions being too close together, and some difficulty in understanding the purpose of the ACT sessions at the beginning. Therapists also indicated some participants were not sure what to expect when commencing the ACT sessions (see Supplement 3 for illustrative quotes).

**Table 4:** Participant self-reported engagement with ACT module materials

Question	*N* (%)	Reasons for non-engagement
Self-reported reading of ACT module booklets		
None of them	2 (7.4)	- Planned to read but never got round to it. - Did not receive.
At least some of them	3 (11.1)	
All of them	14 (51.9)	
Missing	8 (29.6)	
Self-reported listening to audio files		
None of them	1 (3.7)	- Did not receive.
At least some of them	1 (3.7)	
All of them	16 (59.3)	
Missing	9 (33.3)	
Self-reported completion of home-practice tasks—Module 1		
None	1 (3.7)	- Did all exercises but not consistently every day as would have liked, due to struggling with energy/motivation some days. - Completed some but did not have time to do all of it. - Did not receive.
At least some	5 (18.5)	
All of the home practice	12 (44.4)	
Missing	9 (33.3)	
Self-reported completion of home-practice tasks—Module 2		
None	2 (7.4)	
At least some	4 (14.8)	
All of the home practice	12 (44.4)	
Missing	9 (33.3)	
Self-reported completion of home-practice tasks—Module 3		
None	2 (7.4)	
At least some	4 (14.8)	
All of the home practice	12 (44.4)	
Missing	9 (33.3)	

### Fidelity of enactment

Qualitative interview data regarding enactment are summarized, with further details and illustrative quotes available in Supplement 3.

#### SMS

Most participants who received the SMS component reported already having a routine in place to take AET and therefore did not enact the suggestions from the SMS messages. However, women stated the messages would be helpful for anyone without such routines, as the messages often reflected strategies they used. Some participants did still feel the messages were useful as a back-up reminder to take their medication when they had forgotten. Personalizing the timing of the messages was suggested to improve enactment.

#### Information leaflet

Of the participants who recalled receiving the information leaflet, some found the leaflet a helpful point of information to remind themselves why they are taking AET. One participant felt they had already done their own research into AET and therefore did not need further information.

#### Website

A few participants recalled how the website had influenced how they managed their side effects, e.g. through reinforcement of the benefits of a healthy diet and prompting a return to exercise classes to manage AET side effects. However, some participants felt the website did not provide any added information, and others did not experience side effects, so did not enact any suggested strategies.

#### ACT

There were several examples of enactment of the ACT skills, including using mindfulness to improve sleep and hot flushes, revisiting module booklets when struggling generally, engagement in value-based activities, and continuing to use unhooking exercises for difficult thoughts. Barriers to enactment included finding it difficult to put ACT skills into practice, feeling the experience of breast cancer was still too raw, and time constraints limiting daily skill practice.

## Discussion

This process evaluation nested in a pilot optimization trial has demonstrated adequate fidelity of four intervention components aiming to support medication adherence to AET in women with breast cancer. We have provided an exemplar of how to assess multidimensional fidelity, guided by the NIH BCC fidelity framework, across four intervention components in the preparation phase of MOST. Our comprehensive, multimethods approach enabled improvements to be made to the intervention components and trial processes ahead of a planned ORCT [19].

The NIH BCC fidelity framework helped us to identify specific areas with lower fidelity, which can be improved ahead of a full ORCT [26]. Typically, investigators have focused on the fidelity of delivery, which would have limited our learning as the largest reduction in fidelity occurred between delivery and receipt of the components [23]. This was particularly evident in the leaflet and website components whereby some participants could not recall receipt, despite data to support that these components had been delivered. In self-management interventions, fidelity assessments that acknowledge the active role of the participant (e.g. also assessing whether a participant receives and uses an intervention component) are important [23]. The multidimensional fidelity assessment provided a clearer overall understanding of the fidelity of each intervention component.

Our process evaluation benefited from a multimethod approach. The quantitative assessments provided a broad overview while the qualitative assessments offered a chance to explore any barriers to fidelity. For example, the ACT therapists discussed a range of topics that may impact fidelity of delivery (e.g. session length, therapeutic relationship, and stretched NHS resources). Key changes in response to feedback included two of the ACT sessions being extended by 10 min and lower-grade practitioners being able to deliver the sessions (e.g. assistant psychologists/psychological well-being practitioners). Participants were able to provide more in-depth information about barriers to receipt and enactment of the components in the interviews. This led to adaptations including providing more time between ACT sessions and personalizing the timing of SMS messages to improve enactment. A full list of adaptations made as a result of our fidelity assessment is available in Supplement 4.

Fidelity assessments are particularly important when using any experimental design from the factorial family. In a factorial trial, contamination (i.e. when a participant receives a component they were not randomized to receive) across cells can undermine factor effects and be detrimental to the analysis of the trial [15]. To monitor this, we used self-reported data of intervention receipt and identified seven instances of potential contamination. While this method did not definitively establish contamination had occurred, it was an effective way of highlighting potential contamination that could be further explored to reduce the risk of contamination in the future ORCT.

An important consideration in a factorial trial is to ensure each intervention component is not reliant upon the presence of any other intervention component for it to function or make logical sense [15]. Our fidelity of design assessment highlighted that each intervention component targeted distinct BCTs, but also identified overlap between the intervention components. This overlap was deemed unproblematic, as BCTs were operationalized differently across components, meaning the components were still sufficiently distinct, despite targeting the same BCTs. For example, problem-solving was coded for all four intervention components, but each component addresses a different problem to solve (e.g. memory and side effects) and provided different information to do so. At the granularity of BCT level, it would be difficult to develop four intervention components targeting the same overall behavior with no overlap.

Our process evaluation had limitations. In coding the BCTs, we only coded the presence or absence of a BCT, not the dose, which will have varied between BCTs. The use of the newly developed BCT dose ontology could be beneficial in the future [36]. The BCTTv1 does not comprehensively incorporate ACT-based methods (as explained in detail elsewhere [8, 37, 38]), which could explain the lower agreement coefficient for the ACT component. We calculated fidelity of receipt assessments including missing data (i.e. as a percentage of those randomized to the component as opposed to just those who completed the questionnaire) to allow comparisons across components. Missing self-report data do not necessarily indicate lack of adherence, and therefore, actual fidelity of receipt may be higher than reported. For example, of those who responded, 73.7% of participants reported reading all of the ACT module booklets, compared with 51.9% when including missing data. However, several of our assessments were self-reported, which could have biased the findings, particularly as these were completed 4 months postrandomization, and could be subject to social desirability bias. Methods to further improve engagement with self-management interventions and to increase questionnaire response rates to reduce missing data would be beneficial. We were unable to interview participants who had withdrawn from the trial, and all interviewed participants were of White ethnicity. Different barriers to fidelity may be present in those who withdrew and in ethnic minority groups.

In conclusion, we have established adequate fidelity of four intervention components aiming to support medication adherence in women with breast cancer. Based on our results, we have made adaptations to intervention components and trial processes to enhance fidelity prior to an ORCT. We have demonstrated a comprehensive, multimethod assessment of fidelity across multiple dimensions, using the NIH BCC fidelity framework. Our approach to fidelity assessment could act as an exemplar for other investigators undertaking research within the preparation phase of MOST.

## Supplementary Data

Supplementary data is available at *Translational Behavioral Medicine* online.

ibae066_suppl_Supplementary_File_1

ibae066_suppl_Supplementary_File_2

ibae066_suppl_Supplementary_File_3

ibae066_suppl_Supplementary_File_4

## Data Availability

Study data were held securely at the University of Leeds CTRU, and operational processes were defined for the transfer, storage, restricted access, and disposal of personal information. Data will be shared for participants who have consented to use of their data for secondary research and will only be made available in such a way that recipients cannot identify individuals by any reasonable likely means. Data will be shared for projects that are in the public interest and compatible with the original purpose of the data processing. Data access requests should be made to CTRU-DataAccess@leeds.ac.uk.

## Welfare of Animals

This article does not contain any studies with animals performed by any of the authors.

## Transparency Statement

*Study Registration*: Clinical trial registration number: ISRCTN10487576. *Analytic Plan Pre-Registration*: A statistical analysis plan was prepared prior to analysis, but this was not pre-registered. *Analytic Code Availability*: Analytic code may be shared as part of a data access request. *Materials Availability*: Materials used to conduct the study may be available by emailing the corresponding author. However, these are currently being evaluated in a larger trial and may not be available to share until the research is complete.
