# Supplementary material for: Assessing multidimensional fidelity in a pilot optimization trial: A process evaluation of four intervention components supporting medication adherence in women with breast cancer
Source: Transl Behav Med. 2024 Dec 5;15(1):ibae066. doi: 10.1093/tbm/ibae066 (PMC11756324; doi:10.1093/tbm/ibae066)
Supplement: ibae066_suppl_Supplementary_File_1 [file ibae066_suppl_supplementary_file_1.docx]

**Supplement 1: ROSETA conceptual model**

This figure presents the four intervention components and their intended targets to support adherence to AET.

**
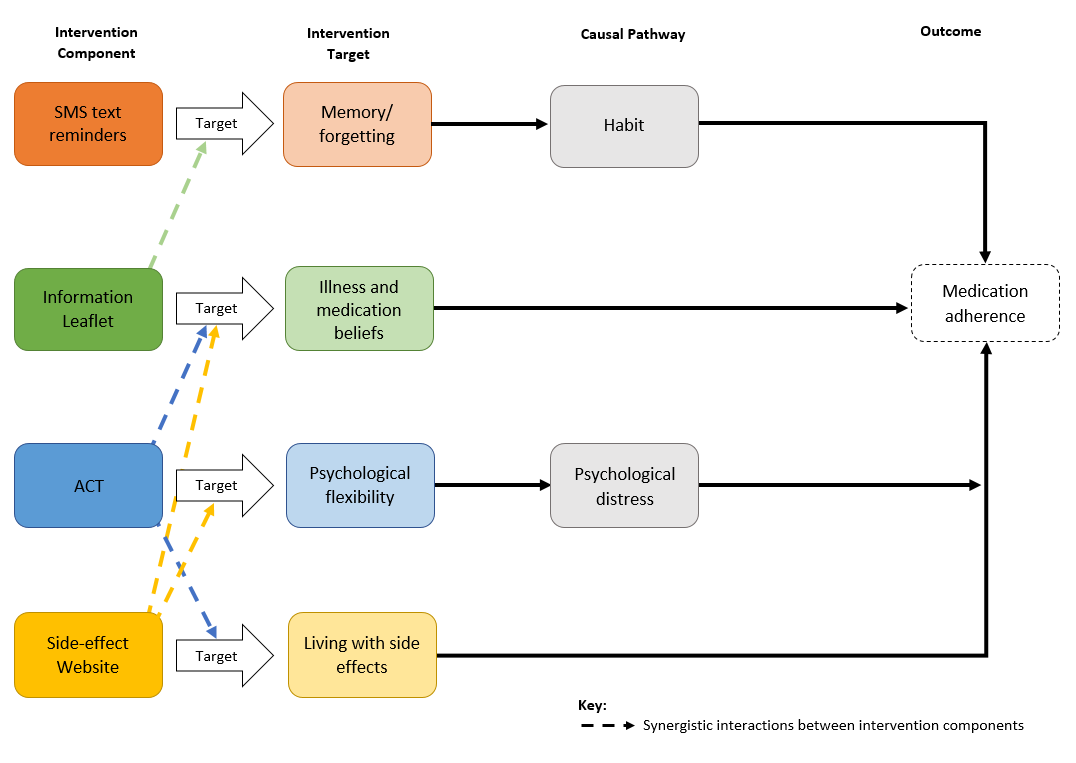
**

Figure taken directly from Green et al., (2022)

Green, S.M.C., et al., *Supporting adjuvant endocrine therapy adherence in women with breast cancer: the development of a complex behavioural intervention using Intervention Mapping guided by the Multiphase Optimisation Strategy.* BMC Health Services Research, 2022. **22**(1): p. 1081.
