# Supplementary material for: Assessing multidimensional fidelity in a pilot optimization trial: A process evaluation of four intervention components supporting medication adherence in women with breast cancer
Source: Transl Behav Med. 2024 Dec 5;15(1):ibae066. doi: 10.1093/tbm/ibae066 (PMC11756324; doi:10.1093/tbm/ibae066)
Supplement: ibae066_suppl_Supplementary_File_2 [file ibae066_suppl_supplementary_file_2.docx]

**Supplement 2: Inclusion and exclusion criteria for the ROSETA optimization trial**

Table taken directly from Smith et al., (2023).

Smith SG, Green SMC, Ellison R, et al. Refining and optimising a behavioural intervention to support endocrine therapy adherence (ROSETA) in UK women with breast cancer: protocol for a pilot fractional factorial trial. *BMJ Open* 2023;13:e069971. doi: 10.1136/bmjopen-2022-069971

| **Inclusion Criteria** | **Exclusion Criteria** |
| --- | --- |
| 1. An informed consent form (signed and dated) 2. Capacity to provide informed consent 3. Women with early stage (1 to 3a) breast cancer according to the TNM / American Joint Committee on Cancer (AJCC) staging system.   *Note. Women being treated for a second primary breast cancer or a breast cancer local recurrence are eligible for the study, providing the most recent cancer is being treated with adjuvant endocrine therapy, and they meet all eligibility criteria. Women with bilateral breast cancer are permitted, providing at least one breast is affected by hormone receptor-positive disease’*   1. Aged ≥18 years at time of screening for ROSETA’s pilot study 2. Have sufficient proficiency in English to be able to adhere to all intervention components and data collection required 3. Treated with curative intent 4. Completed their hospital-based treatment (e.g., surgery, radiotherapy and/or chemotherapy) for the current breast cancer within the last 12 months.   *Note. Women are still eligible for the study if they are being treated with monoclonal antibody-based therapy such as trastuzumab, kadcyla, pertuzumab, and phesgo*   1. Currently prescribed oral adjuvant Hormone Therapy (tamoxifen, raloxifene, anastrozole, letrozole, exemestane) 2. The participant is willing to complete the study questionnaires* 3. The participant is willing to be audio recorded during the therapy sessions* 4. The participant is willing and able to attend all ACT sessions either via video conference or telephone* 5. The participant is willing and able to complete home practice tasks* 6. Access to a mobile phone to receive SMS messages* 7. Willing to receive frequent SMS messages* 8. Access to a computer or smart device that can access the internet* | 1. Stopped taking adjuvant hormone therapy if it is clinically contraindicated according to clinical recommendation 2. Women with Metastatic breast cancer 3. Currently or recently (last 6 months) involved in a similar research study where medication adherence is a primary outcome* 4. Currently attending psychotherapy/psycho-oncology/psychology/counselling services, for any clinical reason* 5. Need for treatment for a severe mental health disorder or crisis, which is likely to interfere with participation (e.g., active psychosis, bipolar disorder, significant issues with addiction or self-harm or expressing active suicidal ideation with active plans and intent*)   *Note, if concerned about the possible presence of risk of suicidal ideation with active plans and intent, then this can be assessed with the following questions, with patients ineligible if they answer ‘yes’ to 5c.*  *Recently (in the last month):*  *a. Have you had any thoughts about ending your life?*  *b. (if yes) Have you thought about how you might go about it?*  *c. (if yes) Do you intend to carry out this plan?*   1. Patients with a scheduled date for breast reconstruction surgery that is within their intervention delivery and follow-up period. *Note: Women planning to have a breast reconstruction but who have not scheduled a date for surgery are permitted* 2. Auditory problems that would prevent the patient from participating in a telephone or video call, or hearing audio clips* |
