# Supplementary material for: Assessing multidimensional fidelity in a pilot optimization trial: A process evaluation of four intervention components supporting medication adherence in women with breast cancer
Source: Transl Behav Med. 2024 Dec 5;15(1):ibae066. doi: 10.1093/tbm/ibae066 (PMC11756324; doi:10.1093/tbm/ibae066)
Supplement: ibae066_suppl_Supplementary_File_3 [file ibae066_suppl_supplementary_file_3.docx]

**Supplement 3- Illustrative quotes for qualitative analysis of interview data per component**

Tables S3a-d provide illustrative quotes for the qualitative assessments of fidelity of receipt and enactment of each intervention component.

| **Table S3a.** SMS component qualitative findings regarding fidelity of receipt and enactment. | | | |
| --- | --- | --- | --- |
| **Fidelity** | **Key findings** | **Description** | **Illustrative quote(s)** |
| Receipt | Reading messages | Most participants reported they always read the messages. | “Even if I didn't look at it straight away, I'd always looking at the same day” (51-69, C5[SMS, ACT]) |
|  | Understanding of messages | Participants generally felt the messages were easy to understand. | “It was simply worded, there was nothing that made you think oh I don’t understand that. You know, whatever state of your education, they were easy, you know, to read and to understand what they were saying.” (≥70, C4[SMS, Web]) |
|  | Barriers to receipt | A minority of participants reported not reading all the messages fully. | “I probably got to the point where I wasn’t reading them, because I would be like oh it’s just the [REDATED] text”. (≤50, C3[SMS, ACT]) |
|  | Opt-out of messages | No interviewed participants opted out, but one participant indicated they may have done outside of a research trial. | “Had I not been within the trial, had, had this been kind of like real life [laughs] if that makes sense, um, but, I may well have done [opted out]… I figured that you know I was in the trial, and therefore I wanted to see what all the messages, because they were clearly different, how they changed and what the messages were like.” (51-69, C2[SMS, IL]) |
| Enactment | Use of messages | Some participants reported using the messages as a back-up reminder. | “I was pretty good in remembering to take me medication, but, the days that, some days when you've got so much on and you forget, that text message was a bit of a lifesaver ‘cos I thought ohh God I have… I haven't.” (≤50, C2[SMS, IL]) |
|  | Barriers to enactment | Most participants reported already having a routine for taking their medication, but often acknowledged the messages could be helpful for others.  Some participants felt the messages were sent at the wrong time of day to be helpful and would find personalizing the time of messages more useful. | “I probably was already using techniques like that, but if I wasn't I think they would have been, er, very helpful. You know, saying try and always take them the same time of the day, that kind of thing, because that is what I do anyway.” (51-69, C5[SMS, ACT])  “I take mine at eight o’clock in the evening, um, because of the, er, side effects…the text message in the morning is great to remind you then, and if you take your tamoxifen then that’s obviously would be a trigger or whatever, but I’ve previously had my phone set for 8 in the evening, so even though I’d had the text message in the morning, it didn’t remind me at 8 o’clock in the evening to take it”. (≤50, C4[SMS, Web]) |
| **Key:** SMS=Short message service. ‘C’= Condition, e.g., C1= Condition 1. Information in square brackets indicates which intervention components participants were randomized to receive: Web= Website component. IL= information leaflet component. ACT= Acceptance and commitment therapy component. AET= Adjuvant endocrine therapy. ≤50= aged 50 or below. 51-69= aged 51 to 69. ≥70=aged 70 or above. | | | |

| **Table S3b.** Information leaflet qualitative findings regarding fidelity of receipt and enactment. | | | |
| --- | --- | --- | --- |
| **Fidelity** | **Key findings** | **Description** | **Illustrative quote(s)** |
| Receipt | Reading leaflet | Some participants recalled receiving the leaflet and reading it at least once or more. | “The usefulness of it for me is that I can always go back to it and read it over again to reassure myself that I’m doing the right thing.” (≥70, C2[SMS, IL]) |
|  | Understanding of leaflet | Participants generally felt the leaflet was easy to understand and was informative. | “It was easy to read, it wasn’t written in complicated medical jargon, so I understood what I was reading. And it wasn’t a load of information bombarded at you. It was, it was concise, it was all I needed to know was in there.” (≥70, C2[SMS, IL]) |
|  | Barriers to receipt | Some participants could not recall receiving the leaflet, often citing that they had received a lot of information. | “Not that I can recall, but I probably did…. I know I got a big e-mail, but I can’t remember, I probably, I got some paperwork, so it probably was on there.” (≤50, C2[SMS, IL]) |
| Enactment | Use of leaflet | Some participants reported re-reading the leaflet every now and then to remind themselves why they are taking AET, and that it is worth trying to cope with the side-effects they are experiencing. | “As I said I get it out every now and again and read through it again because sometimes I think to myself do, I really need to take these tablets and then I go back to it and think well yeah”. (≥70, C2[SMS, IL]) |
|  | Barriers to enactment | One participant felt they had already done their own research into AET. | “So, for me, my, I did a lot of research and reading around it anyway…majority books, um people that I knew, and yeah, research, medical research, um, asking a lot of questions, I probably asked a lot more questions of the nurses and you know key people at the hospital.” (51-69, C2[SMS, IL]) |
| **Key:** SMS=Short message service. ‘C’= Condition, e.g., C1= Condition 1. Information in square brackets indicates which intervention components participants were randomized to receive: Web= Website component. IL= information leaflet component. ACT= Acceptance and commitment therapy component. AET= Adjuvant endocrine therapy. ≤50= aged 50 or below. 51-69= aged 51 to 69. ≥70=aged 70 or above. | | | |

| **Table S3c.** Website component qualitative findings regarding fidelity of receipt and enactment. | | | |  |
| --- | --- | --- | --- | --- |
| **Fidelity** | **Key findings** | **Description** | **Illustrative quote(s)** |  |
| Receipt | Logging in/ reading the website | Many participants reported logging in once but then not revisiting the website. Some participants reported that they did not revisit the website as it was static and so there was no incentive. | “To be honest I didn’t really use them very much. I had one that look, and I might’ve gone back and had another look, um. But um I didn’t really, um I can’t say that I looked at them every week or made an effort to look at them all.” (51-69, C6[IL, Web]) |  |
|  | Barriers to receipt | Two participants could not recall receiving the website log in details. |  |  |
| Enactment | Use of side-effect self-management strategies | Some participants reported specific uses of the website, e.g., to reinforce a healthy diet, and returning to Pilates to help with physical side-effects from AET. | “I think the only thing I did do, was I did, I managed to get back Pilates, and do some Pilates, um, and, um that, that’s good for me, um, because obviously in the class I have to concentrate and focus and do, and do the exercise, and that, it does help with the physical side of things.” (51-69, C7[ACT, Web]) |  |
|  | Barriers to enactment | Some participants felt that there was nothing they didn’t already know on the website.    One participant was not experiencing side-effects. | “I looked at it a couple of times, read through it and thought well I know all of that but it’s not helping me. I think you know, a lot of the things were things I could think of myself, like the gentle exercise and sort of things like that, they were all things I knew, um, and all made sense to me, but I couldn’t seem to, well, sort of couldn’t seem to do them.” (51-69, C7[ACT, Web])  “Maybe I just didn’t feel at that time that I needed any help with anything... I knew it was there if you like, but um, maybe at that time you know I didn’t reach out and feel that I needed, um, I was very inquisitive to begin with but there wasn’t anything that drew me particularly to go back.” (51-69, C6[IL, Web]) |  |
| **Key:** SMS=Short message service. ‘C’= Condition, e.g., C1= Condition 1. Information in square brackets indicates which intervention components participants were randomized to receive: Web= Website component. IL= information leaflet component. ACT= Acceptance and commitment therapy component. AET= Adjuvant endocrine therapy. ≤50= aged 50 or below. 51-69= aged 51 to 69. ≥70=aged 70 or above. | | | | |

| **Table S3d.** ACT component qualitative findings regarding fidelity of receipt and enactment. | | | |  |
| --- | --- | --- | --- | --- |
| **Fidelity** | **Key findings** | **Description** | **Illustrative quote(s)** |  |
| Receipt | Attending sessions | Participants reported being able to attend sessions and valued the flexibility. | “I’m a nurse and I work 3 long days…it worked quite well actually, and ‘cos there was flexibility….when I saw the thing I thought oh is this gonna be like 4 Thursdays in a row or, so you get that kind of oh god I’m gonna have to sort all my shifts out again and, but the fact that it was quite flexible….I never felt there was a pressure around it” (≤50, C3[SMS, ACT]) |  |
|  | Understanding of module materials | Participants reported the booklets and skills were easy to understand. | “I thought it was put together very well, and very sort of easy, you know, easy to understand, the way it was written.” (51-69, C5[SMS, ACT]) |  |
|  | Barriers to receipt | Some participants felt the sessions were too close together and they did not have time to complete home practice.  Some participants reported some difficulty in understanding how the ACT sessions would help them take their medication to begin with. This was also reflected in the therapist interviews. | “I felt like I didn't have much time to sort of do the booklet and work on it before I was having a review...Two to three weeks [between sessions] I would have, I would have felt better. I would have felt like I'd worked on it more and probably could have asked more questions.” (51-69, C3[SMS, ACT])  “I don’t think I fully understood how it linked in and how it helped me adhere to, you know how it would help the study really, I couldn’t really see, get my head around that, but selfishly I felt like it really helped me, so it was really useful.” (≤50, C3[SMS, ACT])  “It was challenging at times…the first participant that I spoke with, um really felt unclear about what this was about, and hadn't expected it to be as personal, um, and so the questions that were in the, in the booklet, um, so this is the idea of it being, you know, psychological therapy. I don't think someone had, really. I I don't know that that had really been conveyed as much to participants.” [Therapist] |  |
| Enactment | Use of ACT skills | Numerous examples were shared regarding using the ACT skills:   - Using mindfulness to help with hot flushes and sleep. - Rereading the booklets to revisit when struggling. - Engaging in values-based activities again e.g., volunteering - Unhooking from difficult thoughts - Managing side-effects of AET | “I didn’t sleep very well…. since I’ve been working on this mindfulness and I haven’t been woken up by hot flushes, I have been sleeping extremely well.” (51-69, C5[IL, ACT])  “I made a lot of notes when I was doing the workbooks and things like that, so I’ve got like notes of stuff to go back to because it’s all very well doing it but if you never, if it never darkens your door again then it’s probably been a bit wasted isn’t it. So, I’ve been, I’ve had like a couple of tricky weeks recently and I’ve kind of gone back over this stuff and tried to like re focus on the skills that I was taught at the time, um, so I think that ongoing that should hopefully be really helpful to me”. (≤50, C3[SMS, ACT])  “I was talking a lot about wanting to do something, but because I I don't have the energy for work, I, I wanted to do you know, something like volunteer and where I only go if I feel up to it, and on Saturday I did a full days volunteer and at, um, our local rugby club, you know and oh my God like it just it's like a new lease of life to be able to go out and spend the day out, my confidence was up to talk to people.” (≤50, C7[ACT, Web])  “I do a lot of unhooking there and then and then I'm like, what do I wanna get out of today and you know, what can I get rid of? What don't I need to carry around with me and it really, it's, it's really working well like it's good.” (≤50, C7[ACT, Web]) |  |
|  | Barriers to enactment | - Difficulty putting skills into practice. - Experience of breast cancer still felt too raw. - Time constraints. | “I found it hard to, to concentrate enough to do them, um, my mind seems to jump around quite a lot, um, and I, I have, I have found it quite hard to concentrate on things in general.” (51-69, C7[ACT, Web])  “Just the opportunity to talk was the most helpful thing. Um, and I could see where it could go with like the, the mindfulness and acceptance that way, I could, I could see where it could go, um, I felt for me it was all too raw still.” (51-69, C7[ACT, Web])  “It's a struggle, isn't it, of, um taking time for yourself and being, and doing all the things that life needs you to do, so it, it wasn't like I didn't do any of them. I definitely did quite a few of them and did quite a lot of thinking around them. But I mean one thing I think I didn't do was, um, you know like literally sit down for 15 minutes every day and do mindfulness.” (51-69, C1[SMS, IL, ACT, Web]) |  |
| **Key:** SMS=Short message service. ‘C’= Condition, e.g., C1= Condition 1. Information in square brackets indicates which intervention components participants were randomized to receive: Web= Website component. IL= information leaflet component. ACT= Acceptance and commitment therapy component. AET= Adjuvant endocrine therapy. ≤50= aged 50 or below. 51-69= aged 51 to 69. ≥70=aged 70 or above. | | | | |
