# Supplementary material for: Assessing multidimensional fidelity in a pilot optimization trial: A process evaluation of four intervention components supporting medication adherence in women with breast cancer
Source: Transl Behav Med. 2024 Dec 5;15(1):ibae066. doi: 10.1093/tbm/ibae066 (PMC11756324; doi:10.1093/tbm/ibae066)
Supplement: ibae066_suppl_Supplementary_File_4 [file ibae066_suppl_supplementary_file_4.docx]

| **Supplement 4- Summary of key adaptations for each intervention component.** | | | |
| --- | --- | --- | --- |
| **Component** | **Suggestion or feedback from participant/therapist** | **Action taken (Y/N)** | **Detail of action taken/ reason why action not taken** |
| SMS | Option to select what time of day messages could be sent | Y | Participants can select messages to be sent morning, lunchtime or evening |
|  |  |  |  |
| SMS | Many women already had routines in place to take their medication | N | Women with all levels of adherence can be recruited as the intervention components could prevent decline of medication adherence, as well as improve low adherence. |
| IL + Web | Some participants did not recall being sent these components; potentially mixed up in large email with other trial documents. | Y | Send leaflet and website components 1 week after randomization, separate to other trial documents. |
| Web | Continuously add new information to the website during the trial, as there is currently no incentive to revisit due to no new information being added. | N | All participants need to have the same experience of the website during the trial. |
| ACT | Weekly sessions were too close together- not enough time to complete home practice. | Y | There is now more flexibility in the timing of the sessions; sessions should be completed within 3 months from the first session and can be delivered weekly or fortnightly within this. |
| ACT | The first and final 15 minute sessions were too short. | Y | All sessions will now be 25 minutes long (10 minutes have been added to the first and final session). |
| ACT | Lower grade practitioners may be able to deliver ACT component | Y | A range of practitioners will now be able to deliver the ACT component, including:   - Health and Care Professional Council (HCPC) registered practitioner psychologist (Clinical, Health or Counselling Psychologist) - UK Council for Psychotherapy (UKCP) registered psychotherapist - Assistant psychologist - Clinical associate psychologist - Individual with formal experience or training in delivering manualized psychological therapy for supporting wellbeing (e.g., a psychological wellbeing practitioner) |
| ACT | Unclear to begin with how ACT could support medication adherence | Y | More information for participants about how ACT could impact adherence, and what is involved in ACT (e.g. you will be expected to reflect on your own thoughts and feelings). |
| SMS: Short message service. IL: information leaflet. ACT: acceptance and commitment therapy. Web: Website. | | | |
